# Anomalously High Recruitment of the 2010 Gulf Menhaden (*Brevoortia patronus*) Year Class: Evidence of Indirect Effects from the *Deepwater Horizon* Blowout in the Gulf of Mexico

**DOI:** 10.1007/s00244-017-0374-0

**Published:** 2017-07-10

**Authors:** Jeffrey W. Short, Harold J. Geiger, J. Christopher Haney, Christine M. Voss, Maria L. Vozzo, Vincent Guillory, Charles H. Peterson

**Affiliations:** 1 JWS Consulting LLC, 19315 Glacier Highway, Juneau, AK 99801 USA; 2 St. Hubert Research Group, 222 Seward, Suite 205, Juneau, AK 99801 USA; 3 Terra Mar Applied Sciences LLC, 123 W. Nye Lane, Suite 129, Carson City, NV 89706 USA; 4 Institute of Marine Sciences, University of North Carolina at Chapel Hill, 3431 Arendell Street, Morehead City, NC 28557 USA; 5 Department of Biological Sciences, Macquarie University, North Ryde, NSW 2109 Australia; 6 296 Levillage Drive, Larose, LA 70373 USA

## Abstract

Gulf menhaden (*Brevoortia patronus*) exhibited unprecedented juvenile recruitment in 2010 during the year of the *Deepwater Horizon* well blowout, exceeding the prior 39-year mean by more than four standard deviations near the Mississippi River. Abundance of that cohort remained exceptionally high for two subsequent years as recruits moved into older age classes. Such changes in this dominant forage fish population can be most parsimoniously explained as consequences of release from predation. Contact with crude oil induced high mortality of piscivorous seabirds, bottlenose dolphin (*Tursiops truncatus*), waders, and other fish-eating marsh birds, all of which are substantial consumers of Gulf menhaden. Diversions of fresh water from the Mississippi River to protect coastal marshes from oiling depressed salinities, impairing access to juvenile Gulf menhaden by aquatic predators that avoid low-salinity estuarine waters. These releases from predation led to an increase of Gulf menhaden biomass in 2011 to 2.4 million t, or more than twice the average biomass of 1.1 million t for the decade prior to 2010. Biomass increases of this magnitude in a major forage fish species suggest additional trophically linked effects at the population-, trophic-level and ecosystem scales, reflecting an heretofore little appreciated indirect effect that may be associated with major oil spills in highly productive marine waters.

Any large oil spill into the ocean typically elicits serious concerns regarding effects on fish. Oil contamination reduces the economic value of harvested fish, and evidence of oil constituents in fish tissues usually indicates more widespread contamination extending to other aquatic taxa. Although the combination of low solubility in water and low mole fractions of the toxic aromatic hydrocarbons in petroleum rarely allow aquatic concentration thresholds for acute toxicity to be attained (>~1 mg/L; Malins and Hodgins 1981; DiToro and McGrath 2000), which accounts for the usual absence of widespread fish mortalities immediately following most oil spills, oil contamination can still have serious population-level effects on fish. For example, ingested oil reduced the growth rate of juvenile pink salmon (*Oncorhynchus gorbuscha*) within the trajectory of the *Exxon Valdez* oil spill in Prince William Sound, Alaska (Carls et al. 1996; Wertheimer and Celewycz 1996; Willette 1996), making them more vulnerable to consumption by predators, and leading to an estimated loss of nearly 28% of the 1989 wild stock year class (Geiger et al. 1996). After the 2007 *Cosco Busan* oil spill, Incardona et al. (2012a, b) attributed high rates of natural spawn mortality of Pacific herring (*Clupea pallasii*) embryos on oil-contaminated shorelines to photo-enhanced toxicity (Arfsten et al. 1996). Confirmation of these adverse effects in the field support expectations that oil contamination from large oil spills generally suppresses survival of exposed fish populations.

Oil spills may also cause adverse effects indirectly, as a result of direct population-level effects on species that have strong ecological linkages to others. For example, extensive oiling of protected rocky intertidal shorelines, often followed by aggressive beach-cleaning efforts after the *Exxon Valdez* oil spill, led to widespread loss of cover normally provided by the rockweed *Fucus gardneri*, and promoted colonization by opportunistic species including green macroalgae and the barnacle species *Chthamalus dalli*, which in turn inhibited and delayed recovery of the normal sheltered rocky shore community (reviewed in Peterson et al. 2003).

Contrary to expectations, evidence supporting suppressed fish populations remained largely absent after the 2010 *Deepwater Horizon* blowout (DWH) in the northern Gulf of Mexico (GoM; Fodrie et al. 2014). Although Gulf killifish (*Fundulus granis*) resident in the immediate vicinity of heavily-oiled coastal marshes displayed adverse effects of oil exposure (Dubansky et al. 2013), populations of juveniles of more than 20 fish species inhabiting seagrass meadows east of the Mississippi River were either unchanged or increased following the DWH as compared with the previous 4 years, with juvenile spotted seatrout (*Cynoscion nebulosus*) increasing by a factor of ~10 in Breton and Mississippi Sounds but not further east (Fodrie and Heck 2011). Schaefer et al. (2016) found that populations of most of the 109 near-coastal fish species inhabiting Mississippi Sound and captured during their sampling increased by a factor of two or more in 2011, and then returned in 2012–2014 to abundances similar to those inferred from methodologically comparable surveys conducted from 1991 to 1994 and from 2000 to 2004.

In particular, Schaefer et al. (2016) found that the abundance of Gulf menhaden (*Brevoortia patronus*), which included young of the year (i.e. age-0) juveniles, increased by a factor of 10 in 2011 compared with the other years considered by their study. This extraordinarily high abundance of Gulf menhaden in Mississippi Sound during 2011 confirms the record-high recruitment indexes for the 2010 year class of Gulf menhaden near the Mississippi River delta reported by the Louisiana Department of Wildlife and Fisheries (LDWF 2011a), which exceeded indices for all prior years since the record began in 1970 by a wide margin. Gulf menhaden are important both economically and ecologically in coastal waters of the GoM near the Mississippi River delta, where they support the second largest commercial fishery in the United States by volume and serve as perhaps the most important forage fish in the waters they inhabit (Geers et al. 2014; Sagarese et al. 2016).

The unprecedentedly high recruitment of Gulf menhaden during the aftermath of the DWH suggests the possibility of intervention by a strong indirect effect of coastal oiling. Fodrie and Heck (2011) and Schaefer et al. (2016) suggest that fishery closures after the DWH contributed to high recruitment and abundance of near-coastal fish in 2010 or 2011. However, offshore spawning of the 2010 Gulf menhaden year class and subsequent advection to estuarine rearing habitat were virtually completed prior to the DWH incident (Shaw et al. 1985; Deegan 1986; Shaw et al. 1988), so it is unlikely that the fishery closures during summer 2010 had much effect on the initial recruiting abundance of the 2010 year class of Gulf menhaden.

Alternatively, an heretofore little recognized possibility that could also contribute to increased abundances of juvenile Gulf menhaden and other near-coastal forage fishes after the DWH is release from predation. Specifically, oil contamination may have induced substantial mortality in species that normally prey heavily on juvenile menhaden, acting in 2010 to release them from typical predatory controls. Explicitly, many of the coastal piscivorous seabird species and bottlenose dolphins (*Tursiops truncatus*) that were killed in large numbers by oil from the DWH (Haney et al. 2014a, Schwacke et al. 2013; Venn-Watson et al. 2015) prey heavily on juvenile Gulf menhaden and other forage fishes, such that their substantially reduced consumption of these juveniles would be evident as increased juvenile survival and hence recruitment of Gulf menhaden.

Here we quantitatively evaluate the biological, environmental and oiling exposure-related factors that may have contributed to the extraordinarily high recruitment of the 2010 Gulf menhaden year class. We rely on the extensive fishery management records of Gulf menhaden recruitment and stock assessments to estimate the likely combined effects of ordinary biological and environmental factors that typically determine recruitment by the end of their first year of life. We compare this estimate to the magnitude of recruitment documented for the 2010 year class, and to estimates of numbers of juvenile Gulf menhaden that would normally have been removed by their predators, especially seabirds, had they not been killed by oil exposure.

## Study Area, Data Sources, and Methods

### Gulf Menhaden Life History and Stock Assessment

**Fig. 1:**
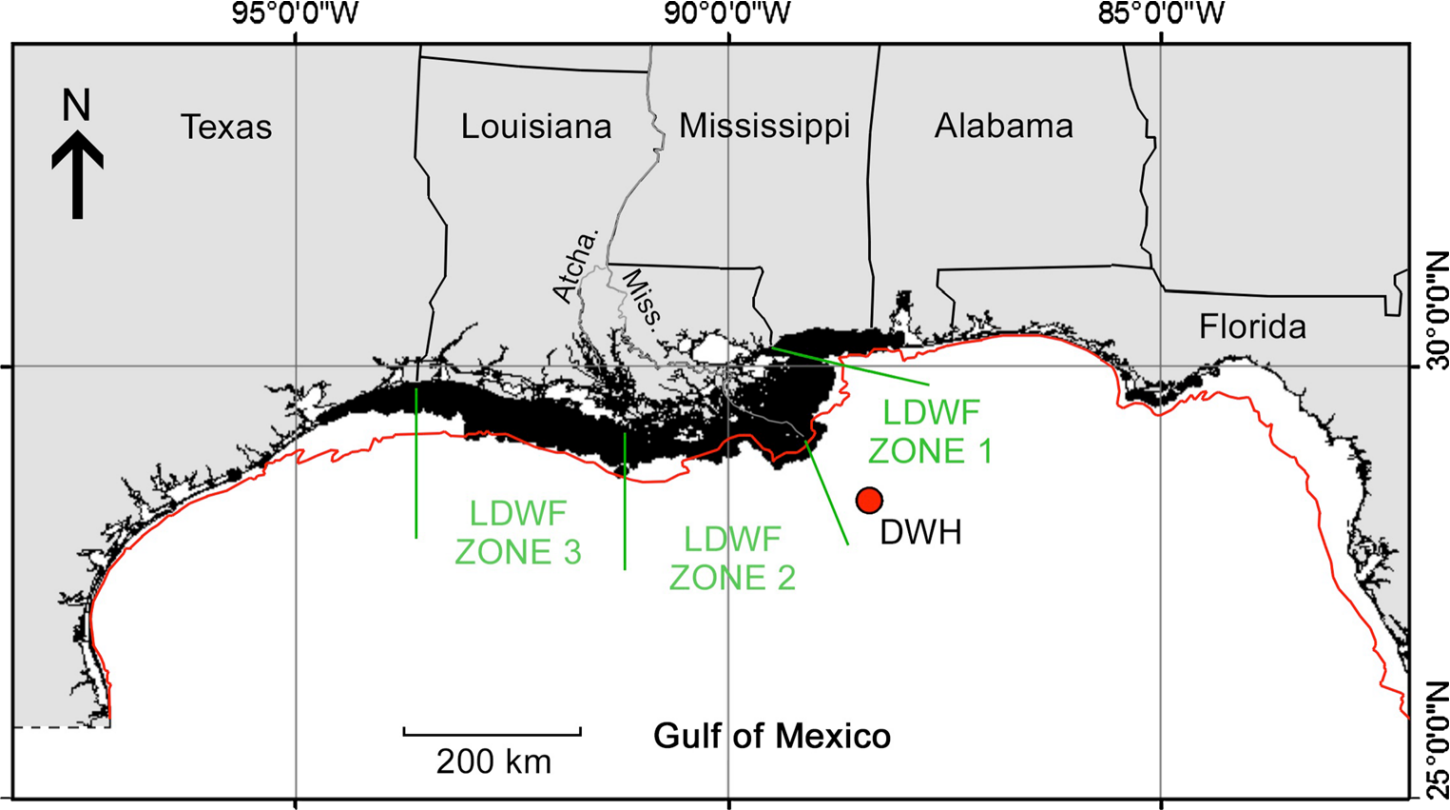
Spatial distribution of commercially exploited aggregations of Gulf menhaden in the northern Gulf of Mexico, as indicated by commercial purse seine fishing effort (*black shading* near shore) during 1986–2011 (from Fig. 5.11 in Schueller et al. 2013). *Red dot* indicates the location of the *Deepwater Horizon* wellhead. Louisiana Department of Wildlife and Fisheries (LDWF) trawl index survey zones are indicated in *green*. The *red line* indicates the 18-m depth isopleth offshore. *Atcha.* Atchafalaya River, *Miss.* Mississippi River

**Fig. 2:**
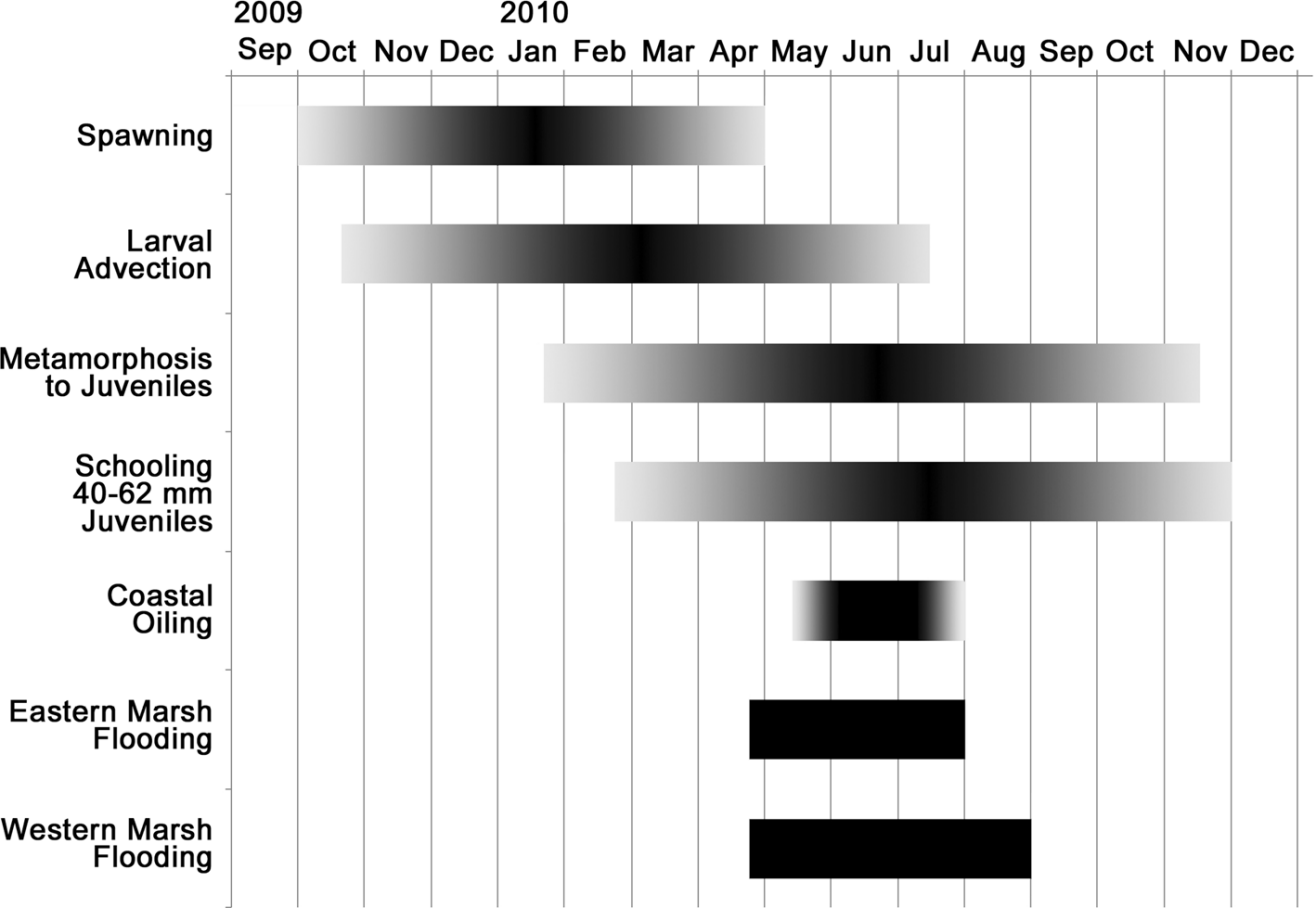
Timing and duration of early life stages of the 2010 Gulf menhaden year class compared with coastal oiling and flooding of the coastal marshes east and west of the Mississippi River following the *Deepwater Horizon* blowout. *Shading in the horizontal bars* represents the gradual increase and decrease of Gulf menhaden spawning activity near the beginning and end of the spawning season and the consequences of the gradual increases and decreases of spawning on the relative magnitudes of larval advection, metamorphosis to juveniles, and schooling of 40–62 mm juveniles; or the gradual increase and disappearance of coastal oiling on the sea surface from the DWH

Gulf menhaden inhabit coastal waters and estuaries of the northern GoM from Mexico to Florida. About 63% of their estuarine nursery habitat is located in Louisiana, especially in the vicinity of the Mississippi River, where most of the Gulf menhaden fishery occurs near shore (Schueller et al. 2013; Fig. 1). Gulf menhaden females release eggs intermittently in multiple batches (Lewis and Roithmayr 1981) throughout a September–April spawning season that peaks during the winter months (Shaw et al. 1985; Fig. 2), and occurs in shallow offshore waters usually less than 18 m deep (Christmas et al. 1982). Fertilized eggs hatch within 2 days (Hettler 1984) and then depend on onshore winds for transport as age-0 larvae, by convention considered born on 1 January of the year identifying a year class, into the fresh or brackish waters in the upper reaches of estuarine nursery habitats within 3–10 weeks (Deegan 1986; Shaw et al. 1988; Fig. 2). Larvae grow about 0.3 mm/day from an initial length of ~2.6 mm (Hettler 1984) and metamorph to juveniles at 30–35 mm length after 3–4 months (Deegan 1986, Fig. 2). Juveniles ~40 mm in length begin migrating to more open waters of estuaries (Deegan 1990, Fig. 2), where they school (Reintjes and June 1961) and graze primarily on phytoplankton and detritus (Olsen et al. 2014). Age-0 juveniles grow exponentially as:

1 $$L(t) = L_{0} \exp \left( {Gt} \right),$$

where *L*_0_ and *G* are respectively 19.50 mm and 0.00485 mm/day (averages of estimates for the 1982 and 1983 year classes in Table 1 of Deegan 1990), and wet weight is allometrically related to standard length as:

2 $$w = aL^{b} ,$$

with *L* in mm, *a* = 8.08 × 10^−6^ and *b* = 3.22 (Deegan 1986).

**Table 1:** Estimates of Gulf menhaden consumption foregone as a consequence of coastal seabirds killed by exposure to crude oil released from the 2010 *Deepwater Horizon* blowout in the Gulf of Mexico

Species	Body mass (g)	Energetic need (kJ/bird/day)	DWH bird loss (#)	Forage fish diet %	Menhaden consumption foregone (g × 10^6^/day)		Prey length (mm)
Northern gannet *Morus bassanus*	3000	3400	35,000	84		**13**	25-305
Laughing gull *Leucophaeus atricilla*	320	680	270,000	45	11		
Herring gull *Larus argentatus*	1200	1700	1600	20	0.07		
Gull sp.	300	650	21,000	45	0.82		
**Total gulls**						**12**	
Brown pelican *Pelecanus occidentalis*	3700	4000	25,000	95	13		60–70
American white pelican *P. erythrorhynchos*	7500	6700	790	50	0.35		≤170
**Total pelicans**						**13**	
Royal tern *Thalasseus maximus*	470	900	31,000	75	2.8		50–100
Sandwich tern *T. sandvicensis*	210	500	24,000	90	1.4		40–80
Least tern *Sternula antillarum*	42	160	39,000	80	0.67		20–90
Forster’s tern *Sterna forsteri*	160	410	15,000	80	0.66		10–100
Common tern *Sterna hirundo*	120	330	13,000	80	0.46		30–130
Caspian tern *Hydroprogne caspia*	660	1100	1100	80	0.13		75–300
Tern sp.	180	450	33,000	80	1.6		
**Total terns**						**7.7**	
**Loons** ***Gavia*** **sp**.	4100	2800	5500	90		**1.8**	30-250
**Black skimmer** ***Rynchops niger***	300	650	14,000	60		**0.73**	30-120
**Grebes** ***Podilymbus/Podiceps*** **sp**.	420	530	5300	50		**0.19**	60-100
**Cormorants** ***Phalacrocorax*** **sp**.	1700	1500	790	50		**0.079**	30-400
**Totals**			**535,000**			**49**	

Bold areas indicate species groups and subtotals. Data rounded to two significant figures, with menhaden foregone consumption aligned on the decimal. See Methods for details on computations

Gulf menhaden mature sexually near the end of their second year as age-1 fish and participate in the offshore reproductive migration. The age-1 and age-2 fish remain in shallow (<20 m) coastal waters during spring and summer (Turner 1969). Although older adult Gulf menhaden along the Louisiana coast have a slight tendency to move towards the Mississippi River delta (Ahrenholz 1981), the river presents a substantial barrier to mixing between sub-populations to its east and west (Kroger and Pristas 1974).

A fishery-independent trawl survey conducted by the LDWF provides the basis for a recruitment index of Gulf menhaden in Louisiana waters. The ~40-station trawl survey started in 1966, with the survey results summarized as an index for each of three zones partitioning the Louisiana coast (Fig. 1), beginning with Zone 2 only in 1970 and then expanded to all three zones beginning in 1973.

The US National Marine Fisheries Service (NMFS) documents the annual Gulf menhaden catch size and samples the catch for length, age and weight determinations. Based on these and other data, the NMFS assesses the Gulf menhaden stock for the whole northern GoM with the Beaufort Assessment Model (BAM), a forward-projecting, age-structured, catch-at-age with additional information (i.e. CAGEAN) model. The BAM uses the Baranov catch equation to determine the effects of fishing and natural mortality on catch, and applies a Beverton-Holt spawner-recruitment relationship (Schueller et al. 2013). Estimated parameters include recruitment to age-0 juveniles, and age- and year-specific fishing mortality and abundance. These parameters are estimated by systematically varying them to match the historical size and age structure of the catch as closely as possible. The natural mortality rates *M*_*a*_, representing the decline of the Gulf menhaden population resulting from losses to predators, diseases, starvation, etc., are not estimated by the BAM but are instead model inputs that have fixed assumed values for each age *a* (i.e. *M*_0_ = 1.62, *M*_1_ = 1.30, *M*_2_ = 1.10, etc.) that remain invariant across years. All of the effects of environmental and ecological variability on Gulf menhaden survival are represented by the age- and year-specific fishing mortality rate estimate, *F*_*a*,*y*_, with value of *F*_0,*y*_, the fishing mortality term for age-0 juveniles, assumed to be zero for all years. The BAM therefore cannot represent inter-annual variability of survival for age-0 juveniles independently, because the estimated total mortality rate for age-0 is determined only by the natural mortality rate *M*_0_. Consequently the BAM recruitment estimates to age-0 are always larger than corresponding recruitment estimates to age-1 the following year by a fixed factor of 5.05 = exp(*M*_0_ = 1.62).

We computed annual average biomass of Gulf menhaden as the product of age-specific abundance reduced by exp(−*Z*_*a*,*y*_/2) to adjust abundance to the middle of the year (where *Z*_*a*,*y*_ is the total mortality rate for age-*a* fish during year *y*, *Z*_*a*,*y*_ = *M*_*a*_ + *F*_*a*,*y*_, *M*_*a*_ from Table 3.11 and *F*_*a*,*y*_ from Table 7.2 in Schueller et al. 2013, and *F*_*a*,2012_ for year 2012 set to the estimate for 2011) and average mid-year weights-at-age of 69.9, 121.6, 168.1 and 205.3 g for age-1, -2, -3 and -4+ fish respectively (from Table 3.10 in Schueller et al. 2013), and 10.5 g for age-0 fish (computed as the time-averaged integral of Eq. 2 over the 274 d that age-0 juveniles grow from average lengths of 35 mm initially to 132.4 mm final length). Our biomass estimate for 2010 is computed on the assumption of actual recruitment to age-0 in 2010 of 174 billion fish instead of 270 billion fish for reasons given in the “Appendix”.

### Deepwater Horizon Blowout

The DWH produced widely transported sub-surface plumes of oil and gas that also included applied chemical dispersants (e.g. Camilli et al. 2010), caused oil contamination of shorelines over a wide span of coastline (Michel et al. 2013; Boufadel et al. 2014), and achieved a cumulative sea surface oil cover of ~120,000 km^2^, as estimated by satellite (e.g. Garcia-Pineda et al. 2012). About 3.3 × 10^5^ m^3^ of South Louisiana crude oil reached the sea surface from the DWH over the course of 86 days (McNutt et al. 2012). Of this amount, an estimated 2.2 × 10^4^ m^3^ of oil (or about 6.7% of the oil that surfaced initially) reached shore, with more than 90% of this deposited on Louisiana shores (Boufadel et al. 2014). The persistence of surface oil slicks was estimated using methods and data sources presented in the supplement to Haney et al. (2014b).

Several thousand water samples were collected by government agencies and analyzed for polycyclic aromatic compounds (PAC), including naphthalene, fluorene, dibenzothiophene, phenanthrene, anthracene, pyrene, fluoranthene, and chrysene, and including alkyl-substituted homologues bearing up to 3 (for fluorene and dibenzothiophene) or 4 (for the others) alkyl carbon atoms, and unsubstituted acenaphthylene, acenaphthene, benz[*a*]anthracene, benzo[*b*]fluoranthene, benzo[*k*]fluoranthene, benzo[*a*]pyrene, benzo[*e*]pyrene, perylene, indeno[1,2,3-*c*,*d*]pyrene, dibenz[*a*,*h*]anthracene, and benzo[*g*,*h*,*i*]perylene. Final results for these compounds were downloaded from http://dwhdiver.orr.noaa.gov, along with the latitude, longitude, depth and date of sample collection. The sum of the concentrations of the PAC listed above are denoted herein as “total PAC”, and are presented as concentration ranges in Fig. 6 for samples collected from the upper 50 m of the water column during the months of May, June and July, 2010.

### Estimation of Seabird Mortality and Foregone Consumption of Gulf Menhaden

Estimates of the numbers of coastal piscivorous seabirds killed are based on those presented in Haney et al. (2014a). Lower estimates presented by the State and Federal trustee agencies for the Natural Resource Damage Assessment (NRDA) of the *Deepwater Horizon* blowout were not used, mainly because the studies on which the NRDA estimates were based prioritized confidence (i.e. low type I error) at the expense of accuracy, which resulted in estimating minimum numbers of seabirds killed instead of the most likely number. The numbers killed for each species of piscivorous seabirds were computed by Haney et al. (2014a) as the number of oiled, dead carcasses of that species recovered from northern GoM shorelines during spring and summer 2010, multiplied by the inverse of the estimated probability that a bird killed by oil would be collected from a shoreline.

We computed the daily consumption of Gulf menhaden separately for each species or species group of piscivorous seabirds. The daily consumption of Gulf menhaden by a species or species group was computed as the ratio of: (1) the product of daily energy requirement for adults of that species or species group, the estimated number of birds of the species or species group that were killed by the DWH, and one half of the dietary proportion of forage fish consumed by the species or species group considered in the numerator; to (2) the product of the energy content of 5.0 kJ/g menhaden wet weight and an assumed assimilation efficiency of 75% (Roby et al. 2003; Brooke 2004) in the denominator. We assumed that Gulf menhaden furnish about half the dietary proportion of forage fish consumed by piscivorous seabirds in the region contaminated by oil from the DWH to allow for consumption of other forage fish species by these seabirds (see below).

The daily energy requirement was computed as log_10_ (daily energy requirement) = *C* + 0.726 log_10_ (kg bird body mass) for the field metabolic requirement of seabirds using *C* = 3.00 for non-flapping flight (loons, black skimmer (*Rynchops niger*), grebes and cormorants), or *C* = 3.19 for flapping flight (all the other seabirds listed in Table 1). This equation is taken from Birt-Friesen et al. (1989), and values for C are from Table 4 therein. Bird body masses are from Sibley (2014), and proportions of forage fish and observed prey lengths in seabird diets were inferred from species accounts in The Birds of North America Online (http://bna.birds.cornell.edu/bna) and references therein.

The average energy content of 5.0 kJ/g wet weight for juvenile menhaden was computed as [(23.64 kJ/g) × (% protein) + (40.16 kJ/g) × (% fat)]/100 from Table 1 in Deegan (1986), which gives percentages of protein and of fat of 16.6 and 2.7% respectively on a wet weight basis for juvenile menhaden averaging 55 mm in length (i.e. about 9 months post fertilization of an age-0 juvenile).

We assumed Gulf menhaden account for about one half of the forage fish consumed by the piscivorous seabirds based on consideration of: (1) the usually turbid waters that limit visual detection of forage fish by avian predators to the uppermost 1 m or less of the water column in Louisiana estuaries, which schools of juvenile Gulf menhaden frequently inhabit (e.g. Deegan 1990); and (2) lower detectability of other abundant forage fish in Louisiana estuaries to avian predators, including bay anchovy (*Anchoa mitchilli*), juvenile Atlantic croaker (*Micropogonias undulatus*), juvenile sand seatrout (*Cynoscion arenarius*), darter goby (*Gobionellus boleosoma*), naked goby (*Gobiosoma bosc*), and other demersal species, either because their peak abundance is achieved during fall or winter, or they do not form schools near the sea surface that would be visible from the air, or they have size ranges that are mostly below 40 mm in length, or they prefer waters farther offshore and at deeper depths, or a combination of these factors, in comparison with juvenile Gulf menhaden (Jones et al. 2002; Baltz and Jones 2003). The diet composition of the one piscivorous seabird species in the region for which detailed studies are available (brown pelican) indicate heavy (up to 95%) dependence on Gulf menhaden (reviewed in Fogarty et al. 1981), suggesting that diets of the other piscivorous species listed in this table may be similarly dominated by Gulf menhaden in response to high detectability of this forage fish when present schooling in the upper 1 m of the water column.

## Results and Discussion

### Recruitment of the 2010 Gulf Menhaden Recruitment Year Class

**Fig. 3:**
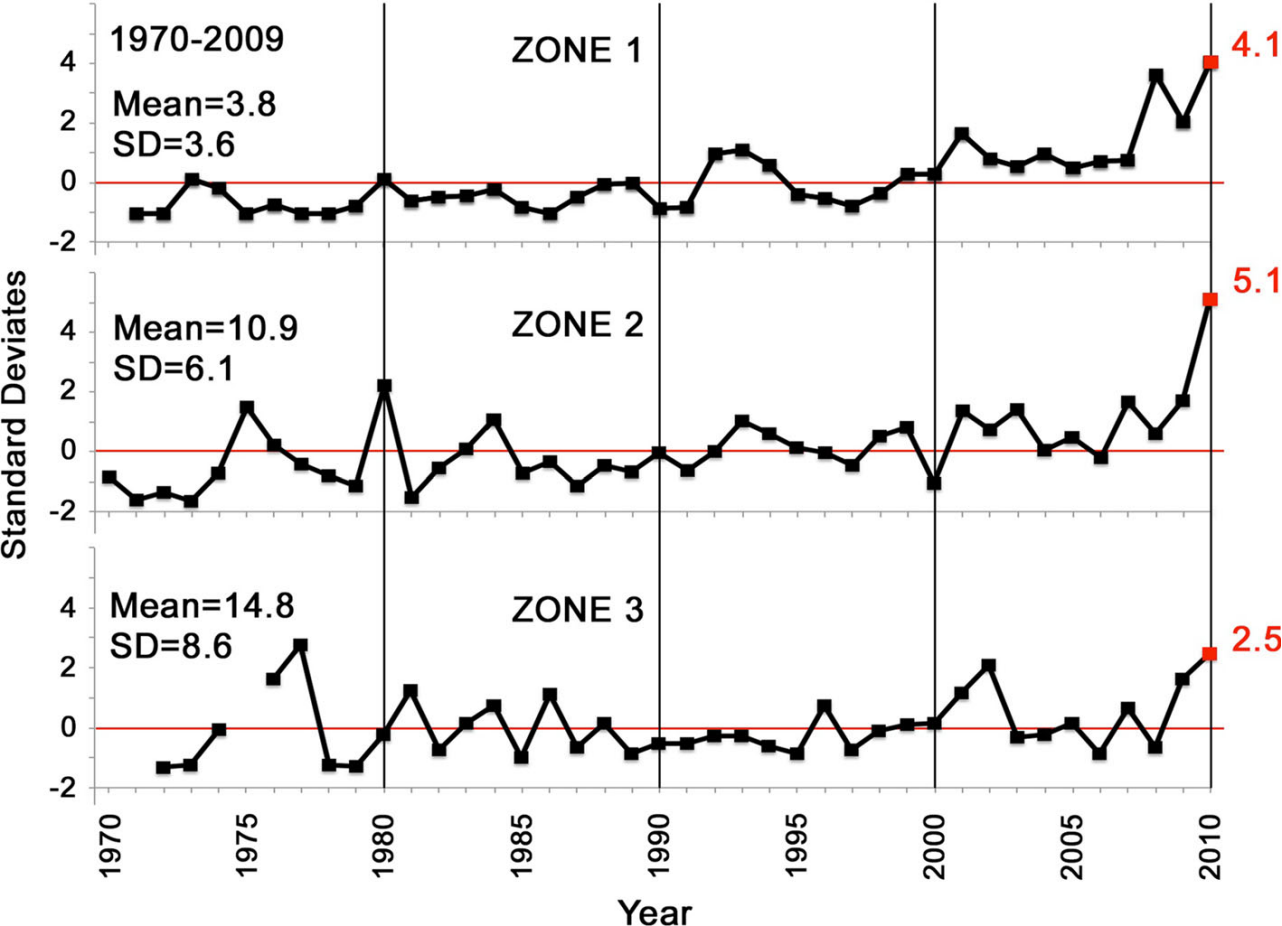
Indices of age-0 juvenile Gulf menhaden recruitment based on LDWF trawl surveys, 1977–2010. *SD* standard deviation. Zone boundaries are indicated in Fig. 1

State and federal monitoring, both fishery-dependent and -independent, consistently indicate unprecedentedly high recruitment of Gulf menhaden to the age-0 juvenile life stage in 2010. The LDWF trawl survey of age-0 Gulf menhaden abundance in LDWF Zone 2 (Fig. 1) between the Mississippi and Atchafalaya Rivers was 5.1 standard deviations greater than the average for the preceding years 1970–2009, and was 4.1 standard deviations above this mean for Louisiana waters east of the Mississippi River in LDWF Zone 1 (Fig. 3). West of the Atchafalaya River in LDWF Zone 3, recruitment was only 2.5 standard deviations greater. Similarly, the NMFS seine index of Gulf-wide age-0 recruitment was almost twice as high in 2010 as any prior year since methodologically consistent multi-state seine monitoring began in 1996 (Table 5.12 in Schueller et al. 2013).

**Fig. 4:**
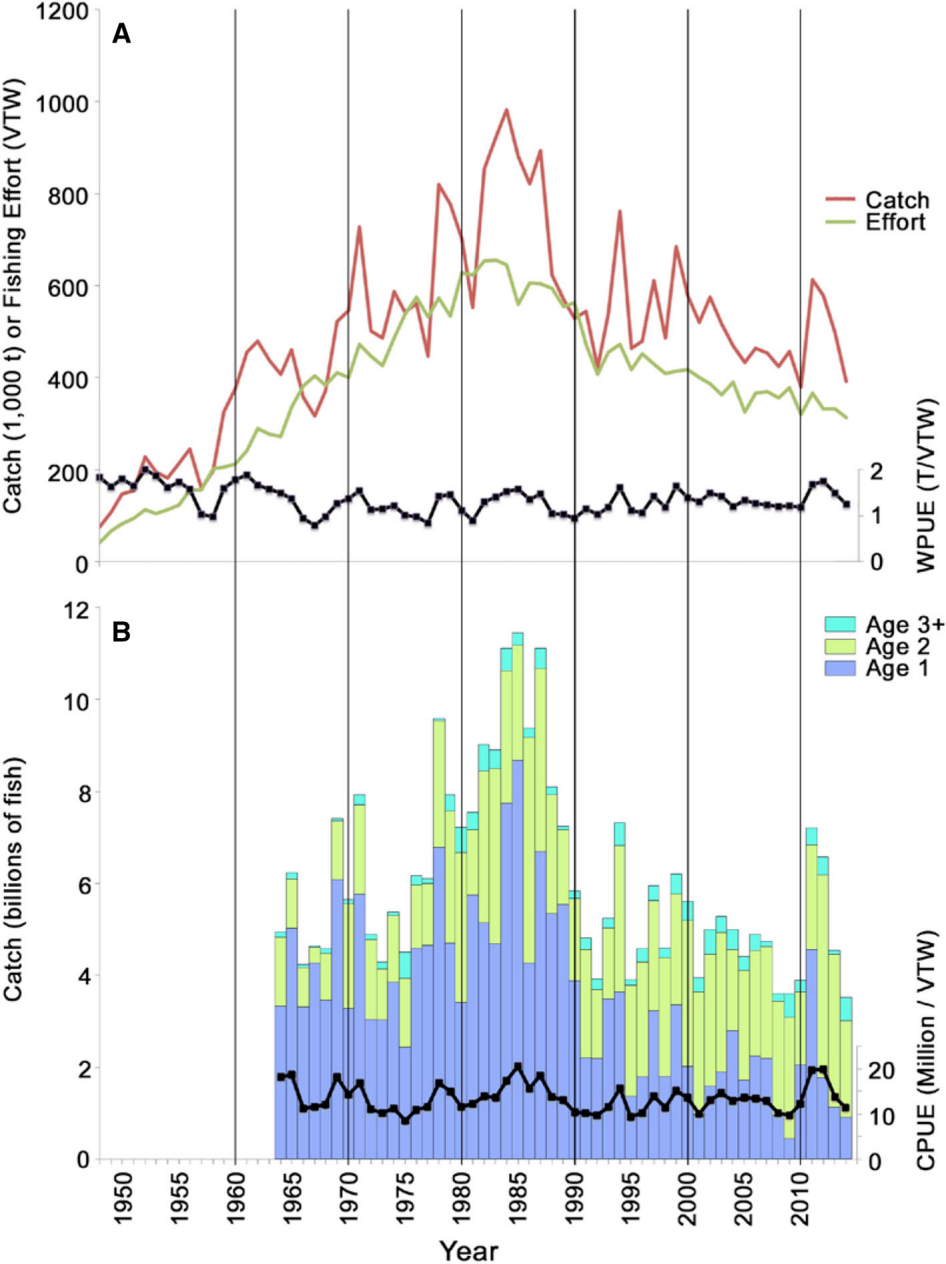
Commercial Gulf menhaden catch: **a** by weight [in millions of tons), effort (as vessel ton-weeks, VTW)], and weight per unit effort (WPUE) as millions of tons/VTW, 1948–2014; **b** by numbers of fish at age, 1964–2014, and catch per unit effort (CPUE) as millions of fish/VTW

Catch monitoring conducted by NMFS confirms the high recruitment of age-0 Gulf menhaden in 2010. The abundance of age-1 fish from the 2010 year class in the 2011 fishery catch was the highest since the late 1980’s despite a decline in fishing effort by a factor of nearly 2 (Fig. 4). High abundance of this year class persisted in the 2012 catch as age-2 fish, the second largest since the record began in 1964, further confirming the high initial recruitment of the 2010 year class and the persistence of this signal over the following 2 years.

**Fig. 5:**
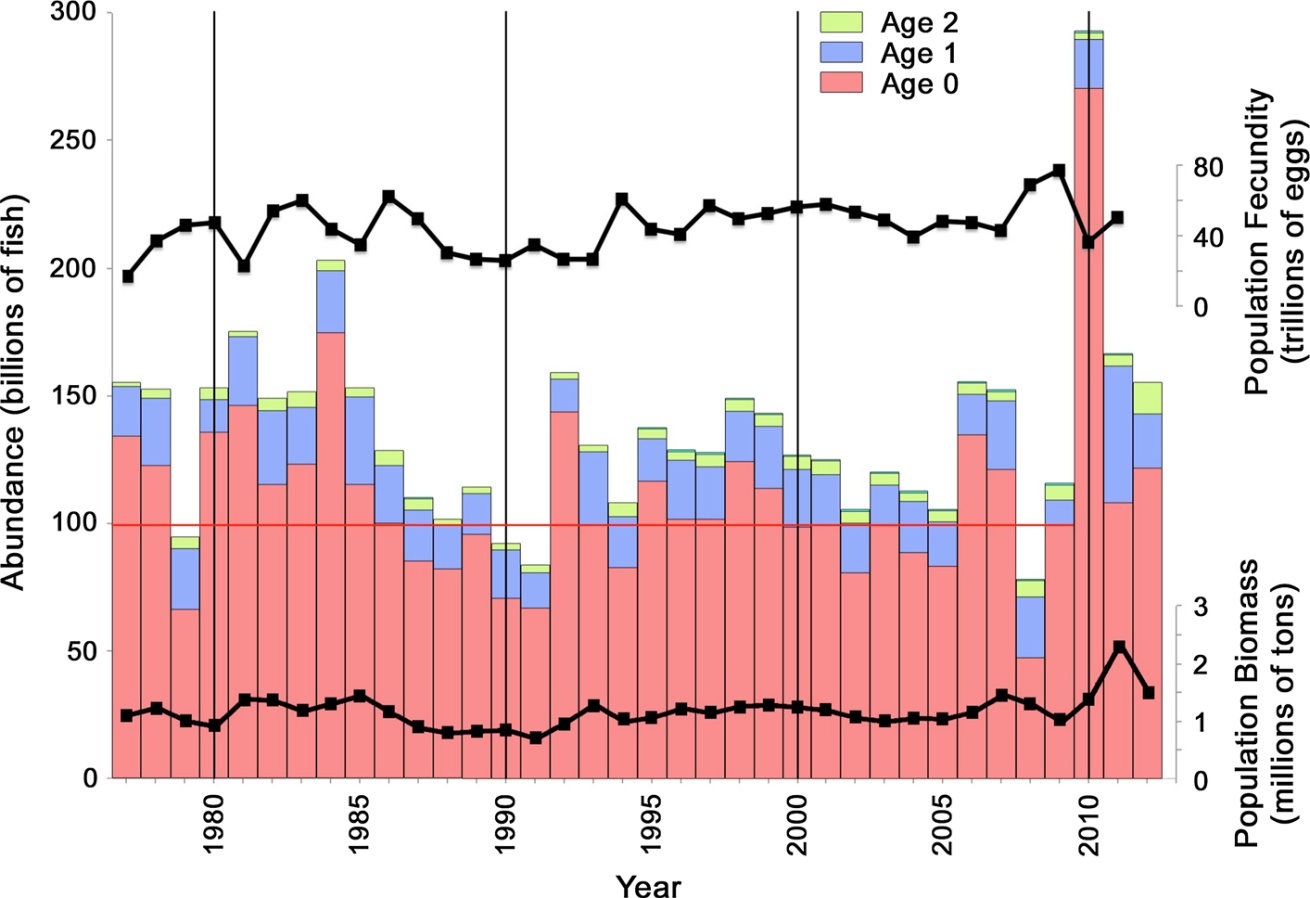
Fecundity, population by age class, and population biomass for Gulf menhaden as estimated by the BAM from 1977 to 2012 (adapted from Fig. 7.11 in Schueller et al. 2013). *Horizontal red line* indicates median (in 1993) recruitment from 1977 to 2009

As integrated by the BAM stock assessment, the monitoring data led to a recruitment estimate of 270 billion Gulf menhaden to the initial age-0 juvenile stage in 2010 (Fig. 5). This is 6.2 standard deviations (1 standard deviation = 26.8 billion fish) above the 105.9 billion average of previous yearly recruitments to the initial age-0 juvenile stage from 1977 through 2009. It is also 3.6 standard deviations, or nearly 100 billion fish, more than the previous record-high recruitment to age-0 of 174 billion fish in 1984 (Fig. 5). The corresponding BAM estimate of age-1 fish in 2011 from the 2010 year class is 53.5 billion fish [=2.7 × 10^10^ exp(*M*_0_ = −1.62)], or 19 billion more fish than the 34.5 billion age-1 fish from the 1984 year class in 1985. Thus, the *increase* of the age-1 population from the 2010 year class over the age-1 population from the 1984 year class, the largest year class prior to 2010 since the record began in 1977, is nearly as large as the entire cohort population at age-1 for a typical year (21 billion age-1 fish; Fig. 5).

### Surface Oiling from the Deepwater Horizon Blowout

**Fig. 6:**
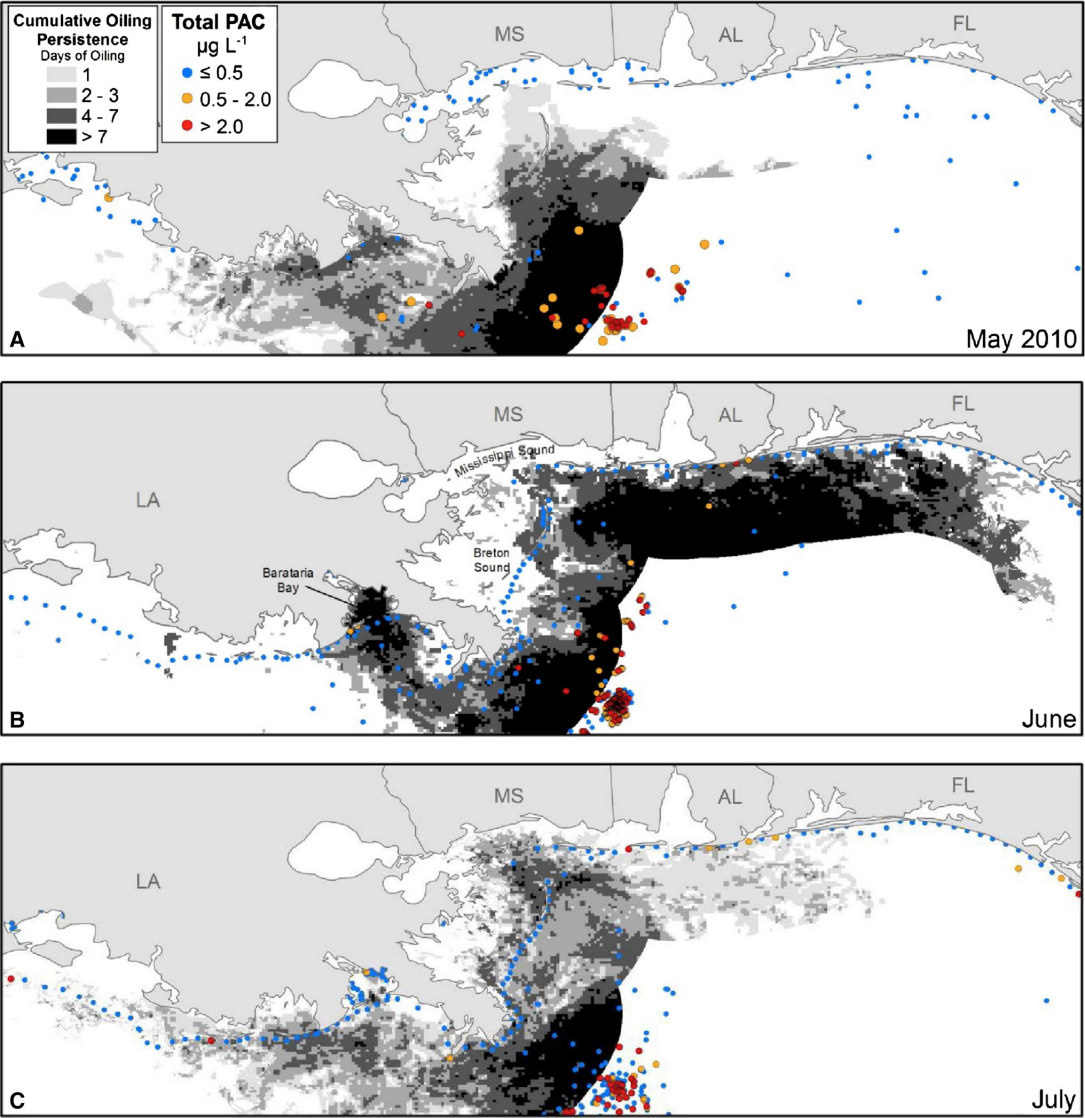
Cumulative oil persistence within 40 km of the shoreline (*grey shading*), and total PAC concentrations in the upper 50 m of the water column (*colored circles*) during **a** May, **b** June and **c** July 2010. The LDWF Zones 1 and 2 are northeast and southwest of the Mississippi River delta, respectively

Variation in the severity of coastal oiling from the DWH blowout along the northern GoM reflects the spatial pattern of Gulf menhaden recruitment strength in 2010. Surface oil slicks were heaviest and most persistent in LDWF Zone 2 (Fig. 1) along the western side of the Mississippi River delta and throughout Barataria Bay (Fig. 6), where oil slicks penetrated the offshore barrier islands resulting in transport into coastal estuaries and marsh edges, contaminating surface waters for weeks. Surface waters in LDWF Zone 1 east of the Mississippi River delta were less heavily but still substantially oiled. Further east, offshore barrier islands protected most of the estuaries and marshes sheltered by them from substantial oiling, and relatively little oil traveled west to the Atchafalaya River or beyond to contaminate coastal estuaries and marshes in LDWF Zone 3 (Figs. 1, 6).

The severity of coastal oiling also reflects the likely spatial distribution of seabirds killed by contact with surface oil slicks. Nearly all of the hundreds of thousands of coastal seabirds estimated to have been killed by contact with oil slicks from the DWH (Haney et al. 2014a) were piscivorous, and the population densities of such seabirds decrease rapidly with distance offshore (Mills 1998; Amorim et al. 2008; Zakkak et al. 2013). Seabird mortalities are thereby predicted to be greatest where the heaviest and most persistent oil slicks intersected with the highest seabird population densities, which occurred along the western side of the Mississippi River delta and shoreward of the coastal barrier island immediately further west, hence especially in Barataria Bay (i.e. LDWF Zone 2; Figs. 1, 6), and immediately east of the Mississippi River delta shoreward of the coastal barrier islands in Breton and Mississippi Sounds (i.e. LDWF Zone 1). In Barataria Bay, heavy oil slicks persisted for weeks (Fig. 6), which may have nearly extirpated seabird populations there given the great sensitivity of seabirds to contact with even small amounts of crude oil (Leighton 1993). More generally, comparison of the spatial variation of seabird mortality inferred from the distribution of coastal oiling intensity and persistence (Fig. 6) with Gulf menhaden recruitment strength in 2010 (Figs. 1, 3) indicates that the two may be closely related.

### Indirect Effects of the Deepwater Horizon Blowout on Gulf Menhaden Recruitment

Removal of seabirds that prey on juvenile Gulf menhaden necessarily increased survival of the juvenile Gulf menhaden. We address the question of how much impact the losses of piscivorous seabirds had on numbers of juvenile Gulf menhaden that would have been consumed had the oiled seabirds remained unaffected by the DWH. Haney et al. (2014a) computed a total of 535,000 (with 95% certainty within about 160,000–1,200,000) dead seabirds that, when alive, prey substantively on forage fish. About half of this total was laughing gulls (*Leucophaeus atricilla*). Other contributing species and species groups include other gulls, pelicans, terns, northern gannets, loons, cormorants, grebes and black skimmers (Table 1).

Based on our computations of the daily energetic requirements of seabirds satisfied through consumption of Gulf menhaden (see Methods), we estimate consumption of about 5 × 10^7^ g wet weight of juvenile Gulf menhaden per day by 535,000 dead seabirds distributed among species according to estimated numbers killed (Table 1). Northern gannets (*Morus bassanus*), laughing gulls, brown pelicans (*Pelecanus occidentalus*) and assorted tern species account for 93% of this consumption, and typically target forage fish ranging from 30 to 100 mm in length.

The estimated numbers of juveniles equivalent to the wet weight of un-consumed Gulf menhaden is sensitive to the assumed size selectivity of the seabird predators for their forage fish prey, and the vulnerability of fish within the preferred size ranges. Juvenile Gulf menhaden spend about 3 months from the time they begin forming schools in open estuarine waters at about 40 mm in length (Deegan 1986) to reach 62 mm in length (Eq. 1). The range of the Gulf menhaden spawning season and the subsequent developmental time ranges leads to metamorphosis to juveniles from about mid-April through mid-August, with the bulk of schooling 40–62 mm juveniles present from about mid-May through mid-September (Fig. 2). Juvenile Gulf menhaden schools are probably most vulnerable to avian predation during the initial 2 or 3 months after forming schools in the more open estuarine waters, when their schools are most readily visible from the air in the shallow estuarine waters. Note that the initial formation of these juvenile Gulf menhaden schools in large numbers coincided closely with the onset of widespread seabird mortalities from coastal oiling in 2010 (Fig. 2), so that the piscivorous seabirds that would have heavily targeted these fish schools were rapidly diminished just as the schools became widely exposed to avian predation.

If consumption of Gulf menhaden were mainly satisfied by juveniles 40–62 mm in length when they are initially exposed to predation by coastal seabirds, an average weight of 2 g for this size range of Gulf menhaden (computed as the time-averaged integral of Eq. 2 over the time required for length to increase from 40 to 62 mm) implies a daily consumption of about 2.5 × 10^7^ fish [=(5 × 10^7^ g)/(2 g/fish)]. This suggests that oil-caused mortality of 535,000 seabirds may have increased survival of juvenile Gulf menhaden by ~2.5 × 10^7^ fish/day, or by a total of ~5 billion fish [=(2.5 × 10^7^ fish/day)(200 days)] over the course of 6+ months from the onset of bird mortalities in mid-May through late November of 2010 (Fig. 2).

Foregone consumption of ~5 billion juvenile Gulf menhaden in the coastal area by the estimated 535,000 seabirds killed by the DWH is ecologically feasible given the abundance of age-0 juveniles during a typical year. The assumed natural mortality rate *M*_0_ = 1.62 implies that, for an average year, the initial recruitment of 105.9 billion fish at metamorphosis to the age-0 juvenile life stage (Fig. 5) declines to 21 billion juveniles [=105.9 billion fish × exp(−1.62)] at the beginning of age-1 the following year, indicating that 85 billion age-0 juvenile Gulf menhaden (i.e. 105.9 billion–21 billion) are either consumed by predators, or die from diseases, exposure to hypoxic conditions, or other factors that contribute to the natural mortality rate. This suggests that consumption of age-0 Gulf menhaden by all avian predators combined, including those not accounted for in Table 1 and those that survived the DWH, could range into the tens of billions during a typical year and still only account for a modest fraction of the ~85 billion age-0 Gulf menhaden that are removed in total. Such a substantive role played by seabird predation on a forage fish population is not unique, as it has been noted previously for the anchoveta (*Engraulis ringens*) fishery off Peru (Schaefer 1970).

We underestimate enhanced survival of juvenile Gulf menhaden in the coastal area by excluding (1) avian predation on fish smaller than 40 mm in length, (2) the increase in energy demands to provision seabird chicks, and (3) predation foregone by bottlenose dolphins and other piscivorous marine mammals killed by exposure to oil (Schwacke et al. 2013; Venn-Watson et al. 2015). Perhaps more importantly, we did not consider reduced predation associated with un-quantified yet evident, oil-induced mortality among the piscivorous birds inhabiting the extensive marshes and estuarine shorelines associated with the Mississippi River delta. Marsh-dwelling birds, such as herons, egrets, rails, bitterns, and cormorants, may prey heavily on small juvenile menhaden, but their population losses from oil contact were not well quantified. The combined additional consumption of juvenile Gulf menhaden by seabirds to meet demands to provision chicks, by bottlenose dolphins and other piscivorous marine mammals that were killed, and by marsh-dwelling birds could increase substantially the actual survival of age-0 Gulf menhaden to well above the ~5 billion fish we estimate on the basis of the oiled seabird carcasses retrieved from shorelines.

Depressed salinities that resulted from diversion of Mississippi River water to coastal marshes to reduce oiling from the DWH may have been another source of indirect effects that decreased predation mortality of juvenile Gulf menhaden. The State of Louisiana dramatically increased the flow rates out of Mississippi River freshwater diversions into Barataria Bay and Breton Sound to impede the flow of oil into upper estuary marshes. Freshwater flow to these marshes increased by a factor of ~5 from late April through late July for eastern marshes or through early-September 2010 for western marshes (O’connor 2013; Fig. 2). The salinities of marshes east of the Mississippi River remained below 10‰ out to the open waters of Breton Sound during May and June 2010 (LDWF 2010), and below 6‰ west of the Mississippi River to western Barataria Bay from June through early September 2010 (LDWF 2011b). These diversions also correspond with the period of greatest abundance of 40–62 mm age-0 Gulf menhaden (Fig. 2). These abnormally low salinities provided enlarged areas of effective shelter for juvenile Gulf menhaden from predation by marine fishes and invertebrate predators that usually consume juvenile Gulf menhaden but prefer higher salinities (Das et al. 2012) contributing to increased survival and hence recruitment of Gulf menhaden to age 1.

Our estimate of increased survival on the order of 5 billion or more juvenile Gulf menhaden serves primarily to demonstrate that the deaths of hundreds of thousands of coastal seabirds would certainly have increased survival of age-0 Gulf menhaden substantially, feasibly by several billions of fish. Greater precision in such estimates is unwarranted given the considerable uncertainties associated with the numbers of coastal seabirds killed, their actual daily energy requirements, their dietary dependence on Gulf menhaden, prey size selectivity, and dates of onset of mortality of birds that regularly prey on juvenile Gulf menhaden. In any case a survival increase of even ~5 billion age-0 juveniles in the coastal area, based only on the oiled seabird carcasses retrieved from shorelines, is sufficiently large to be considered as substantive in comparison with recruitment of 21 billion juveniles to age-1 during a typical year (Fig. 5).

### Other Factors Affecting Gulf Menhaden Recruitment in 2010

Compared with many other forage fish species, the population of Gulf menhaden had been extraordinarily stable prior to the DWH, varying in abundance by less than a factor of 3 from 1977 to 2009 (Fig. 5). Population fecundity determines the initial abundance of Gulf menhaden larvae, while marine feeding conditions and speed of physically driven transport to estuarine rearing habitat determine initial survival. Subsequent survival in estuaries as larvae grow and metamorph to juveniles varies with availability of food supplies and oxygen, and with losses to predation. In 2010, direct interactions arising from the toxic effects of crude oil and dispersants following the DWH must also be considered as factors potentially affecting recruitment.

Weak correlation between fecundity and recruitment, as estimated by the BAM from 1977 through 2009 (*r* = −0.251, *df* = 31, *P* = 0.159; from data presented in Tables 7.4 and 7.5 in Schueller et al. 2013; plotted in Fig. 5), indicate that Gulf menhaden recruitment is mainly determined by factors other than fecundity. Estimated Gulf menhaden fecundity for the 2010 year class was 36.4 trillion eggs, about 81% of the average fecundity of 45.1 trillion eggs for 1977–2009, which should have led to near-average recruitment in 2010 if recruitment were determined mainly by fecundity.

**Fig. 7:**
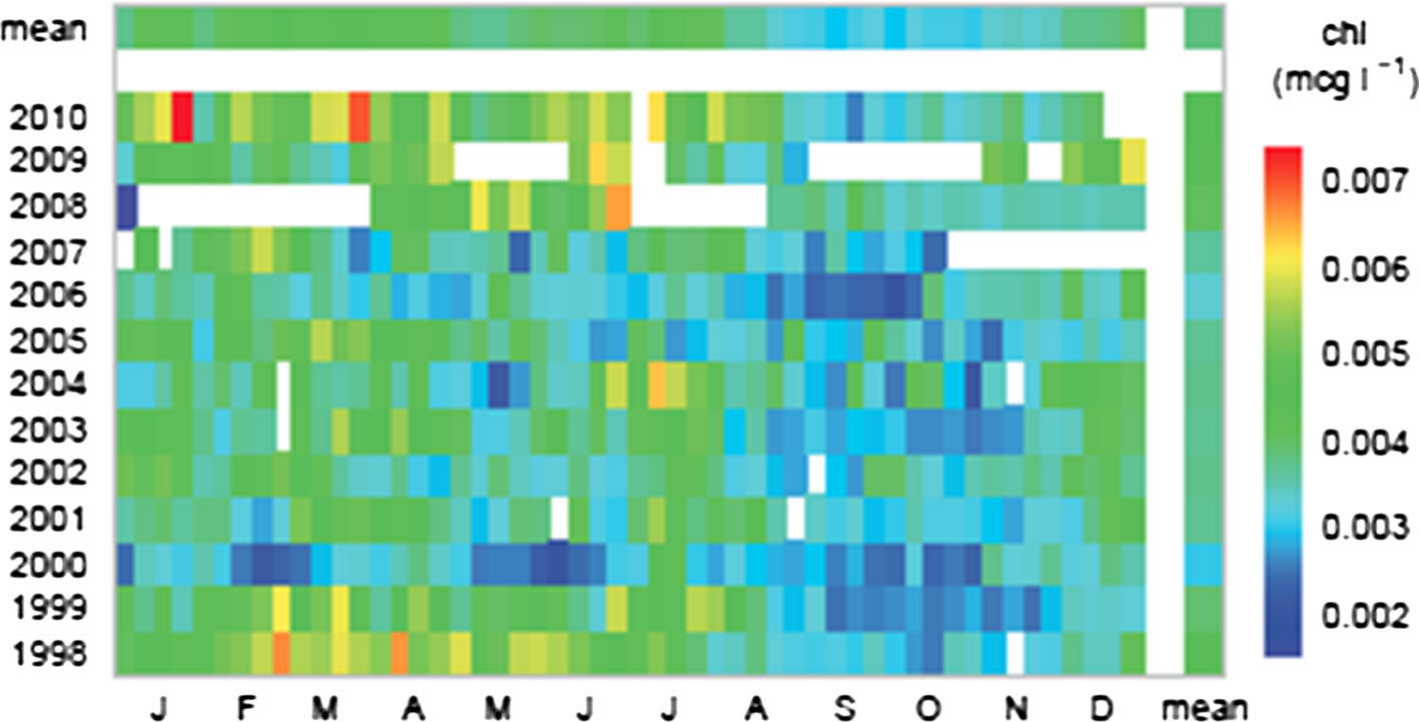
Monthly median chlorophyll *a* calculated from Sea-viewing Wide Field-of-View Sensor (SeaWiFS) satellite imagery in coastal northern Gulf of Mexico surface waters integrated between −87°W and −93°W latitude and from seafloor depths between 10 and 100 m (from Karnauskas et al. 2013)

Marine feeding conditions were exceptionally favorable for survival of newly-hatched Gulf menhaden larvae in 2010. Monthly median chlorophyll *a* calculated from Sea-viewing Wide Field-of-View Sensor (SeaWiFS) satellite imagery was ~50% greater during January through April 2010 than in the decade prior (Fig. 7, from Karnauskas et al. 2013) in coastal waters from 10 to 100 m seafloor depth and between −87°W and −93°W latitude, which encompasses most of the Gulf menhaden spawning habitat. Similarly high and persistent chlorophyll *a* concentrations were detected by SeaWiFS imagery in 1998 in these waters (Fig. 7; see also Fig. 12 in Muller-Karger et al. 2015), but this was associated with only modestly greater-than-average recruitment of age-0 Gulf menhaden for that year (Fig. 5). Comparison of age-0 recruitment for 2010 and 1998 with corresponding SeaWiFS chlorophyll *a* concentrations suggests that while high concentrations of phytoplankton available to Gulf menhaden larvae at sea enhance recruitment, the elevated concentrations present in 2010 apparently account for only a small part of the exceptional increase in recruitment that occurred that year.

**Fig. 8:**
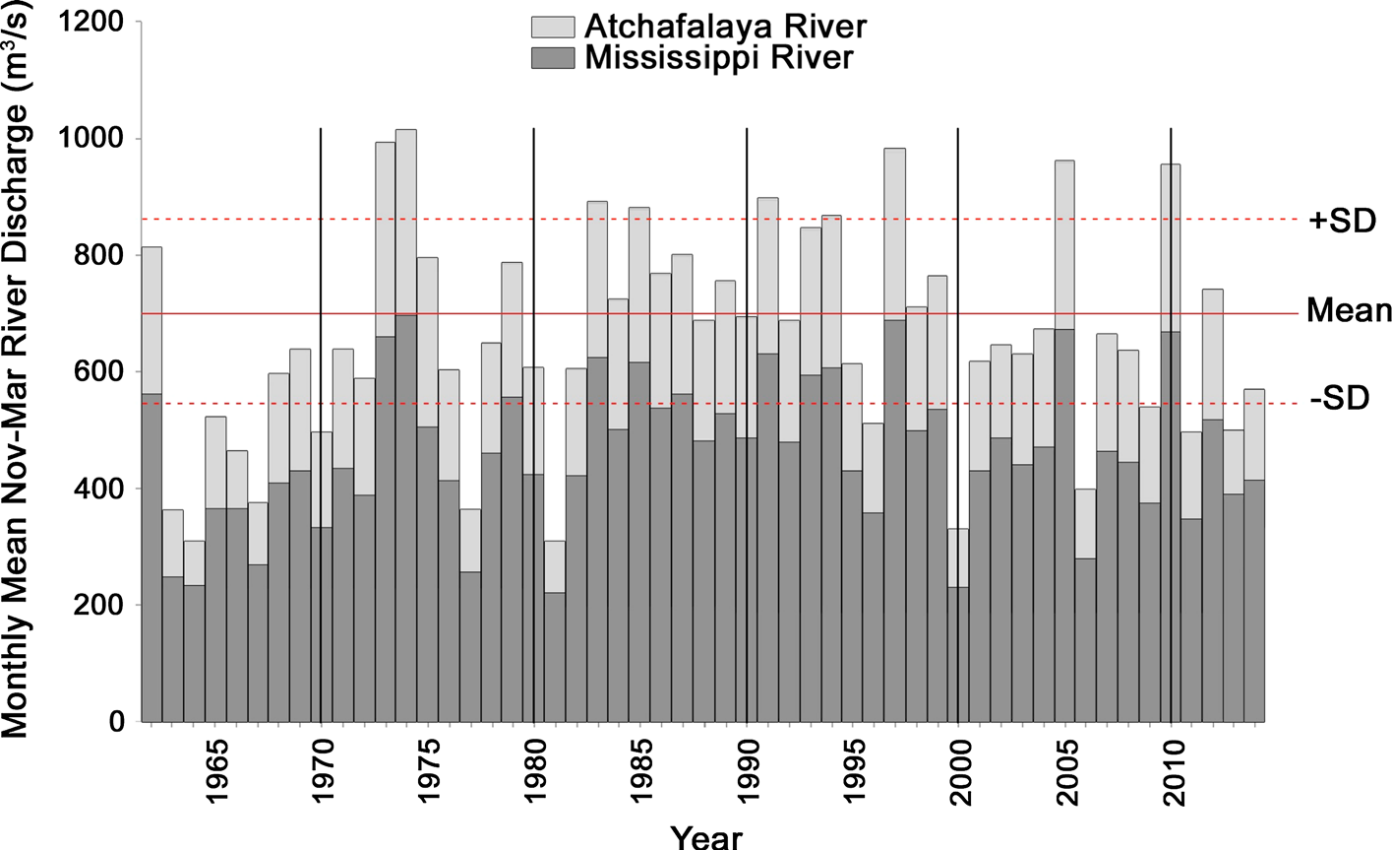
Mean monthly discharge of Mississippi and Atchafalaya Rivers, November through March, 1962–2014. *SD* standard deviation. Discharge data downloaded on 2 October 2015 for US Army Corps of Engineers gaging stations at Tarbert Landing, LA from rivergages.mvr.usace.army.mil/WaterControl/shefgraph-historic.cfm?sid=01100Q for the Mississippi and at Simmesport, LA from rivergages.mvr.usace.army.mil/WaterControl/shefgraph-historic.cfm?sid=03045Q for the Atchafalaya

The oceanographic conditions that led to higher neritic phytoplankton concentrations during winter and spring 2010 also had the consequence of impeding passive transport of Gulf menhaden larvae to their estuarine rearing habitat. The high neritic phytoplankton concentrations during winter 2010 were caused by unusually high discharge from the Mississippi River (Fig. 8) combined with unusually strong and persistent offshore winds. High river discharge during fall and winter promotes surface layer stratification of receiving waters by less dense river water laden with inorganic nutrients essential for phytoplankton growth (Lohrenz et al. 1997), which led in 2010 to an intense winter phytoplankton bloom over an unusually large area adjacent to the Mississippi River delta (Fig. 7; Huang et al. 2013). The strong, persistent offshore winds during winter and spring (Huang et al. 2013), together with high river discharge during fall and winter, impeded larval transport from offshore towards the estuarine rearing habitat. Govoni (1997) found that annual Mississippi River discharge was negatively associated with numbers of half-year recruits, and suggested that higher discharges result in an expansive plume, which may propel larvae further offshore and delay the shoreward transport of larvae, hence increasing their vulnerability to predation. Historically, mean monthly discharges of the Mississippi and Atchafalaya Rivers from November through March accounted for nearly 30% of Gulf menhaden recruitment variability in regression analyses (Guillory et al. 1983; Vaughan et al. 2011). Hence, the oceanographic conditions during winter and spring 2010 should have lowered recruitment of the 2010 Gulf menhaden year class, partially or even entirely offsetting the effects of favorable phytoplankton abundance.

Schools of juvenile Gulf menhaden experience large fish kills in shallow estuarine waters during exceptionally hot weather, which contribute to natural mortality (VanderKooy and Smith 2002). Cool summers may therefore be expected to promote the recruitment of age-1 juveniles. However, as measured by the number of days when the maximum air temperature at New Orleans exceeded 35 °C, 2010 was the 4th hottest summer since 1977, thereby increasing the likelihood of large fish kills caused by estuarine hypoxia and hence reducing recruitment.

The seasonal succession of habitats occupied by the 2010 year class of Gulf menhaden ensured that direct effects of exposure to crude oil released during the DWH or to dispersants released subsequently were virtually negligible. By the time the blowout occurred on 20 April, the offshore spawning of the 2010 year class was complete and nearly all of the larvae had been transported to brackish upper-estuarine rearing habitat, removing larvae away from possible exposure to crude oil or dispersants (Fig. 2). Juvenile Gulf menhaden from the earliest spawning of the 2010 year class in fall 2009 would have begun forming schools and occupying more open and saline estuarine waters by March or April, where they did not encounter oil until these habitats became oiled initially in mid-May. Similarly, juveniles from later spawns would have first encountered oil in the open estuarine waters for a few weeks at most during June and early July. Exposure to contaminants derived from dispersants or from crude oil from the DWH was therefore limited to intermittent or transient occupancy of waters beneath surface oil slicks for a few days or weeks by a small part of the 2010 year class of Gulf menhaden, even in heavily oiled embayments such as Barataria Bay, because surface slicks were widespread in these embayments for less than about 2 of the 6+ months of outmigration of juveniles from their brackish upper-estuarine rearing habitat.

Direct toxicity of crude oil or dispersants that were associated with the DWH to age-0 juvenile Gulf menhaden, or to the piscivorous fishes that consume them was also probably negligible, because of the low aqueous solubility of toxic components in crude oil or dispersants and the great dilution capacity of the receiving surface waters. Toxicity thresholds depend on mode of toxic action, ranging from narcosis induced mainly by mono- and dicyclic aromatic compounds at concentrations near one part per million (Malins and Hodgins 1981; DiToro and McGrath 2000), to embryotoxic and photo-enhanced toxicity induced by certain PAC at less than one part per billion (Carls et al. 2002; Duesterloh et al. 2002; Mager et al. 2014). However, contaminant monitoring after the DWH provides little support for inferring toxic effects of oil to aquatic organisms present in the nearshore water column, except in the immediate vicinity of shorelines that were heavily and persistently oiled. The overwhelming majority of surface seawater samples taken from the uppermost 50 m near shore during 2010 failed even to detect PAC at detection limits in the low parts per trillion (Fig. 6). Deployment of polyethylene membrane passive sampling devices along the coast of the northern GoM before and after shoreline oiling from the DWH confirm these results (Allan et al. 2012). Total PAC concentrations inferred from these deployments were consistently less than 0.03 µg/L at all but one station (Grand Terre, LA), where concentrations approached 0.2 µg/L during early summer 2010 but declined to <0.03 µg/L by August.

### Comparison of Ordinary Factors that Typically Affect Gulf Menhaden Recruitment with Indirect Effects of the Deepwater Horizon Blowout

Ordinary biological and environmental factors that typically affect Gulf menhaden recruitment, such as changes in population fecundity, early life stage feeding conditions, speed of transport to estuarine rearing habitat, etc., alone or in combination, fail to adequately account for the extraordinarily high recruitment of juvenile Gulf menhaden documented in 2010. Prior to 2010, the highest recruitment of age-0 Gulf menhaden estimated by the BAM in 1984 led to an estimate of 34 billion age-1 Gulf menhaden at the beginning of 1985 (Fig. 5). The 2.5 standard deviation increase of age-0 recruitment observed in the 2010 LDWF trawl surveys west of the Atchafalaya River (Fig. 3), which was much less affected by oiling from the DWH, suggests the magnitude of the increased recruitment in 2010 that may be attributed to the combined effects of the ordinary biological and environmental factors that typically affect recruitment. Given that average recruitment to age 1 from 1977 through 2009 was 21 billion fish with a standard deviation of 5.3 billion (Fig. 5), 2.5 standard deviations above the mean amounts to a population size at age-1 of 34 billion fish, similar to the previous record set by the 1984 year class. Recruitment of age-0 Gulf menhaden in 1998, when larval feeding conditions offshore were nearly as favorable as in 2010 (Fig. 8), led to 24 billion age-1 Gulf menhaden in 1999. Given that favorable larval feeding conditions offshore in 2010 were at least partially offset by unfavorable conditions for larval transport onshore to estuarine nursery habitat, it seems unlikely that even an unusually favorable combination of ordinary biological and environmental factors that typically affect recruitment would have led to an age-1 population much greater than 34 billion fish from the 2010 year class.

The 2010 Gulf menhaden year class had an estimated 53.5 billion age-1 fish, or ~19 billion more than the ~34 billion age-1 fish we consider near the maximum that could result from the combined effects of ordinary biological and environmental factors. This additional ~19 billion age-1 fish above the highest previously-estimated recruitment on record (in 1984, see Fig. 5), is nearly equivalent to the median recruitment of 21 billion age-1 fish from 1977 to 2009, and hence is, by itself, nearly as large as the entire recruitment of Gulf menhaden during a typical prior year. Of this ~19 billion increase of age-1 fish, decreased predation caused indirectly by the DWH may well have been the major contributing cause. This increased survival of several billions from decreased predation does not imply unreasonably high numbers of seabirds killed, of juvenile Gulf menhaden that would ordinarily be consumed by them, or unfeasibly large proportions of natural mortality losses implied by the foregone consumption. Although our assumptions led to an estimated additional survival of ~5 billion juvenile Gulf menhaden in the coastal area because of the piscivorous seabirds killed by the DWH, overall survivals could have been considerably higher if more seabirds were killed, or the seabirds killed preferred to consume smaller sizes of juveniles or smaller sizes were more available to them, or juvenile Gulf menhaden account for substantially more than 50% of forage fish consumed by these seabirds during summer and fall. Recognizing the additional numbers of juvenile Gulf menhaden that were not consumed to provision the chicks of the seabirds killed, or that would have been consumed by the bottlenose dolphins or piscivorous birds inhabiting marshes that were killed by exposure to oil, along with possibly reduced predation by aquatic predators that avoid low-salinity estuarine waters, suggests to us that most or even all of the ~19 billion additional surviving age-1 juvenile Gulf menhaden may have been an indirect consequence of direct effects of the DWH on predators of juvenile Gulf menhaden.

Conversely, ordinary biological and environmental factors that typically affect Gulf menhaden recruitment, alone or in combination, fail to provide an obvious explanation for the strong variation of Gulf menhaden recruitment along the Louisiana coast in 2010, where recruitment was strongest where coastal oiling was heaviest and most persistent (Fig. 6), whereas oil-caused mortalities of coastal seabirds and marine mammals account for this variation readily. While it is always possible that some unknown combination of factors may in fact be responsible for the extraordinary recruitment of juvenile Gulf menhaden, we could find no evidence that substantively contradicts the effects we have ascribed to release from predation caused by the DWH, and we do find considerable circumstantial evidence in support. We therefore conclude that mortalities of seabirds, marsh birds and marine mammals, perhaps augmented by habitat exclusion of aquatic predators intolerant of low salinities, was a major factor explaining the anomalously large recruitment of the 2010 year class of Gulf menhaden.

Our evaluation of the potential causes of spatially explicit increases in recruitment of Gulf menhaden following the DWH provides compelling evidence for a little appreciated environmental effect that may be generally induced by large oil spills. Actually, this effect of increased fishery recruitment may have been observed but not recognized many times before in fishery populations after major oil spills, with the analysts looking at the affected populations failing to observe this signal when mixed in with large process errors in computing recruitment. Following the DWH, the increases of numerous fish species reported by Fodrie and Heck (2011) and by Schaefer et al. (2016) may also have resulted mainly from reduced predation from piscivorous seabirds killed by exposure to DWH oil. We arbitrarily assumed that juvenile Gulf menhaden furnished 50% of the forage fish diet of the piscivorous seabirds listed in Table 1, the other 50% being furnished by other species of forage fishes. Hence, our estimate of increased survival on the order of ~5 billion age-0 Gulf menhaden in the coastal area implies a survival increase of comparable magnitude for the other forage fish species that would have been consumed by piscivorous seabirds had they not been killed by oil from the DWH.

This process whereby recruitment of Gulf menhaden and of other forage fish species is enhanced in nearshore nursery habitats may have had consequences at the ecosystem scale in the northern Gulf of Mexico. The increased recruitment of the 2010 year class of Gulf menhaden led to an increase of population biomass of nearly 1.3 million t, or more than twice the average biomass of 1.1 million t for the decade prior to 2010. An increase of this magnitude in a forage fish species that is already a major component of the neritic ecosystem of the northern GoM (Geers et al. 2014; Sagarese et al. 2016) raises the possibility of additional effects associated with trophic linkages to Gulf menhaden at population-, trophic-level and ecosystem scales. Substantially greater biomass of Gulf menhaden would increase predation on their planktonic prey, which could conceivably affect recruitment of other aquatic species that co-inhabit waters occupied by Gulf menhaden. Greater biomass of Gulf menhaden in 2011 (Fig. 5) also increased their availability to their surviving predators, presumably benefiting their recovery. Analyses of the dynamics of interacting species in this study suggest that indirect effects of large oil spills may be much more important, more subtle and wide-reaching than has been previously appreciated.

## Appendix

Our assumption that actual recruitment of age-0 Gulf menhaden in 2010 was 174 billion fish in our computations of Gulf menhaden biomass for the years 2010 in Fig. 5 is motivated by our recognition that the BAM is not capable of representing inter-annual variation in age-0 survival. The BAM recruitment estimate to age-1 of 53.5 billion fish in 2011 necessarily implies a corresponding estimate of 270 billion age-0 fish in 2010, because these two estimates are strictly related by the factor 5.05 [=exp(*M*_0_ = 1.62)]. Alternatively, the 53.5 billion age-1 fish estimated for 2011 more probably resulted from an initial age-0 abundance that was lower than 270 billion fish in 2010 but had higher survival to age-1 than is determined by the assumed natural mortality rate of *M*_0_ = 1.62, because of the reduced predation that resulted from the DWH in 2010.

We assumed that the actual recruitment of age-0 Gulf menhaden in 2010 was 174 billion fish because: (1) this was the highest age-0 recruitment estimated by the BAM (in 1984) from 1977 to 2009; and (2) the increase in the recruitment index for age-0 Gulf menhaden in the LDWF Zone 3 was 2.5 standard deviations above the mean prior to 2010 and beginning in 1972 (Fig. 3), and assuming the recruitment of age-0 juveniles for the entire population would have been 2.5 standard deviations above the long-term mean had the DWH not occurred leads to an estimate of 173 billion juvenile age-0 Gulf menhaden at the beginning of 2010. That is, the average recruitment to age 1 from 1977 through 2009 was 21 billion fish, with a standard deviation of 5.3 billion fish (Fig. 5), so 2.5 standard deviations above the mean of 21 billion fish is 21 billion + 2.5 × 5.3 billion = 34.3 billion fish, and (34.3 billion fish) [exp(1.62)] = 173 billion age-0 juveniles. The near agreement of this result computed from the long-term BAM estimate of age-1 recruitment prior to the DWH and its standard deviation, with the maximum age-0 recruitment ever recorded during the same time interval, suggests to us that the actual recruitment of age-0 Gulf menhaden in 2010 was probably much closer to 174 billion fish than to 270 billion fish. We used the value of 174 billion age-0 Gulf menhaden in our biomass computations because using the 270 billion fish as estimated by the BAM would have excessively inflated our biomass estimates for 2010.
